# Hydroxyurea-Stalled Replication Forks Become Progressively Inactivated and Require Two Different RAD51-Mediated Pathways for Restart and Repair

**DOI:** 10.1016/j.molcel.2010.01.021

**Published:** 2010-02-26

**Authors:** Eva Petermann, Manuel Luís Orta, Natalia Issaeva, Niklas Schultz, Thomas Helleday

**Affiliations:** 1 Gray Institute for Radiation Oncology and Biology, University of Oxford, Oxford OX3 7DQ, UK; 2 Department of Cell Biology, University of Seville, E-41012 Seville, Spain; 3 Department of Genetics Microbiology and Toxicology, Stockholm University, SE-106 91 Stockholm, Sweden

**Keywords:** DNA

## Abstract

Faithful DNA replication is essential to all life. Hydroxyurea (HU) depletes the cells of dNTPs, which initially results in stalled replication forks that, after prolonged treatment, collapse into DSBs. Here, we report that stalled replication forks are efficiently restarted in a RAD51-dependent process that does not trigger homologous recombination (HR). The XRCC3 protein, which is required for RAD51 foci formation, is also required for replication restart of HU-stalled forks, suggesting that RAD51-mediated strand invasion supports fork restart. In contrast, replication forks collapsed by prolonged replication blocks do not restart, and global replication is rescued by new origin firing. We find that RAD51-dependent HR is triggered for repair of collapsed replication forks, without apparent restart. In conclusion, our data suggest that restart of stalled replication forks and HR repair of collapsed replication forks require two distinct RAD51-mediated pathways.

## Graphical abstract

**Graphical Abstract:**

**Figure:**
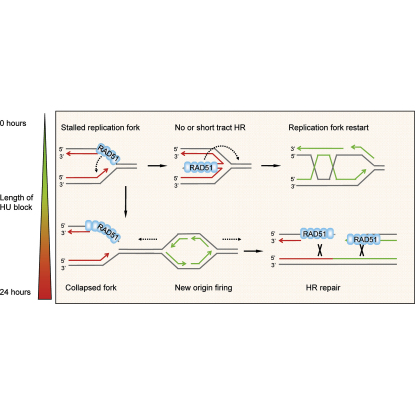

**Highlights:**

► RAD51 promotes replication fork restart after short hydroxyurea (HU) blocks ► RAD51-mediated fork restart does not create homologous recombination (HR) products ► Long HU blocks lead to fork collapse; replication is restarted by new origin firing ► Collapsed replication forks are repaired by classical RAD51-dependent HR

## Introduction

Faithful DNA replication is essential to maintain genome integrity and prevent the accumulation of cancer-promoting mutations. Replication forks encounter numerous obstacles on the DNA template, which can lead to fork stalling or fork collapse—that is, the dissociation of the replication machinery and the generation of replication-dependent DNA double-strand breaks (DSBs). The restart of stalled replication forks is best characterized in *Escherichia coli*, where reactivation is essential because of the lack of backup origins. Forks in *E. coli* are reactivated by recombination-dependent or -independent pathways catalyzed by the RuvABC or PriA and PriC proteins, respectively (Heller and Marians, 2006). These proteins are not conserved in eukaryotes, and the extent and mechanisms of eukaryotic replication fork reactivation are not well characterized. In mammalian cells, agents that stall or collapse replication forks, such as hydroxyurea (HU), thymidine, and camptothecin, strongly induce homologous recombination (HR), which promotes the survival of these treatments (Arnaudeau et al., 2001; Lundin et al., 2002; Saintigny et al., 2001), suggesting that recombination-dependent replication restart mechanisms might also be used by higher eukaryotes. If forks are kept stalled for more than 12 hr, increasing amounts of fork-associated DSBs are generated (Saintigny et al., 2001) in a process dependent on the structure-specific endonuclease MUS81 (Hanada et al., 2007). This supports a model of replication fork restart via recombination, initiated by a one-ended DSB, which is similar to the RuvABC-mediated mechanism (Helleday, 2003; Heller and Marians, 2006). MUS81-dependent DSBs only start to appear after many hours of HU treatment, and the formation of RAD51 foci at stalled forks is independent of DSB formation (Hanada et al., 2007), suggesting that RAD51 might be involved in a different fork restart mechanism avoiding DSB formation, especially after short replication blocks (Helleday, 2003). RAD51, the eukaryotic RecA homolog, is an essential HR factor that catalyzes homology search and strand exchange (Baumann et al., 1996; Li and Heyer, 2008). RAD51 promotes survival of replication stress and prevents accumulation of replication-associated DSBs (Lundin et al., 2003; Sonoda et al., 1998). The formation of RAD51 presynaptic filaments and resulting nuclear foci in response to HU is mediated by the RAD51 paralogs, including XRCC3 (Bishop et al., 1998).

Here, we analyze replication restart after different lengths of HU blocks and the roles played by RAD51 in this process. Our data suggest that RAD51 has distinct early and late roles during replication blocks, facilitating replication fork restart when forks are still viable and repairing fork-associated DNA damage after forks have collapsed and global replication is rescued by new origin firing.

## Results

### Replication Forks Become Inactivated during Prolonged Replication Blocks

**Figure 1:**
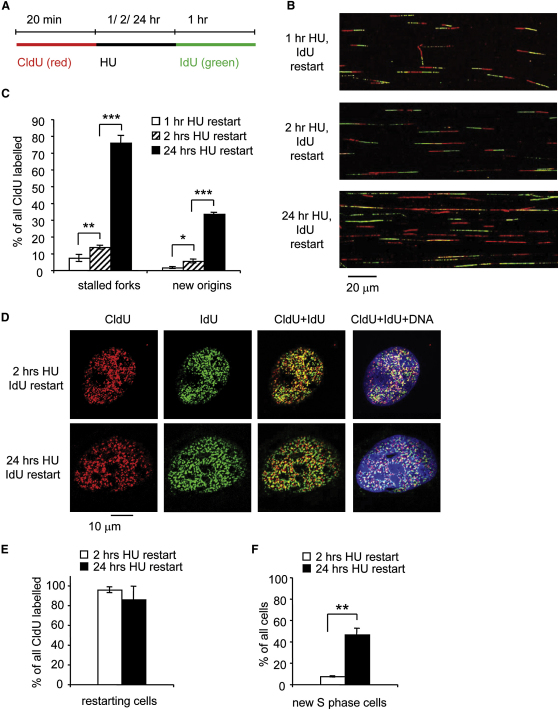
Replication Forks Restart after Short Replication Blocks but Become Inactivated during Long Replication Blocks (A) Labeling protocols for DNA fiber and replication foci analysis. U2OS cells were pulse-labeled with CldU, treated with 2 mM HU for the times indicated, and released into IdU. CldU was detected using a specific primary antibody and a secondary antibody in red. IdU was detected using specific primary antibody and a secondary antibody in green. (B) Representative images of replication tracks from U2OS cells after release from 1, 2, or 24 hr HU treatment. (C) Quantification of fork restart in U2OS cells after release from 1, 2, or 24 hr HU treatment. (D) Global replication restart in U2OS cells after release from 2 or 24 hr HU treatment. Cells were pulse-labeled as in (A), then fixed and immunostained for CldU (red) and IdU (green). DNA was counterstained with DAPI (blue). (E) Quantification of cells restarting global replication as in (D). Cells labeled with both CldU and IdU are shown as percentages of all cells labeled with CldU. (F) Quantification of cells newly entering S phase after release from 2 or 24 hr HU treatment. Cells labeled with only IdU are shown as percentages of total cells. The means and standard deviation (SD) (bars) of at least three independent experiments are shown. Values marked with asterisks are significantly different (Student's *t* test, ^∗^*p* < 0.05, ^∗∗^*p* < 0.01, and ^∗∗∗^*p* < 0.001; see also Figure S1).

To understand the fate of replication forks following replication blocks, we analyzed the restart of replication forks after different periods of HU treatment using the DNA fiber technique (Henry-Mowatt et al., 2003). HU depletes deoxyribonucleotide pools and immediately stalls replication forks (Bianchi et al., 1986). U2OS cells were pulse-labeled with 5-chlorodeoxyuridine (CldU) for 20 min, washed and blocked in HU for 2 hr, washed again and pulse-labeled with 5-iododeoxyuridine (IdU) for 1, 2, or 24 hr (Figure 1A). Afterward, DNA spreads were prepared and analyzed by immunofluorescence (Figure 1B). To quantify replication fork restart, the amount of stalled forks was related to the total number of replication tracks labeled with CldU (Figure 1C). Surprisingly, we found that, although most forks restarted after release from a 1 or 2 hr HU block, most forks remained stalled after release from 24 hr HU block. Instead of restarting forks, replication tracks labeled only with IdU appeared that seemed to result from new initiation events (Figures 1B and 1C). Because it was previously shown that nucleotide incorporation resumes between 12 and 18 hr of HU blocks (Hanada et al., 2007), we included IdU during the HU treatment to determine whether the apparent lack of restart was due to forks moving large distances during the treatment, resulting in the two labels becoming separated (see Figure S1A available online). Most forks moved less than 6 μm (15.5 kb) during the 24-hr block (Figures S1B and S1C). CldU tracks in less than 6 μm distance from IdU tracks were therefore not considered as stalled forks. Taking this into consideration, our data suggest that most forks become inactivated after long times in HU.

### Replication Restarts by Firing of New Origins after Long Replication Blocks

To test whether the new initiation events observed during restart from 24 hr HU blocks occurred in cells that had been replicating before the HU block, or only in cells newly entering S phase, cells were pulse-labeled like before, but then fixed and immunostained to reveal replication foci (Figure 1D). Most cells that had contained active forks before the HU treatment did resume replication after removal of the drug (Figure 1E). As expected, a large number of cells also newly entered S phase and were only labeled with IdU (Figure 1F). These observations suggest that most of the replication restart observed after release from long HU blocks does not involve elongation of existing forks, but instead the firing of new replication origins. Fork inactivation and new origin firing after 24 hr HU treatment was not dependent on the processing of stalled forks into DSBs, because both were not affected by siRNA depletion of MUS81 (Figures S1D–S1F).

### Replication Forks Accumulate Damage after Long Replication Blocks

**Figure 2:**
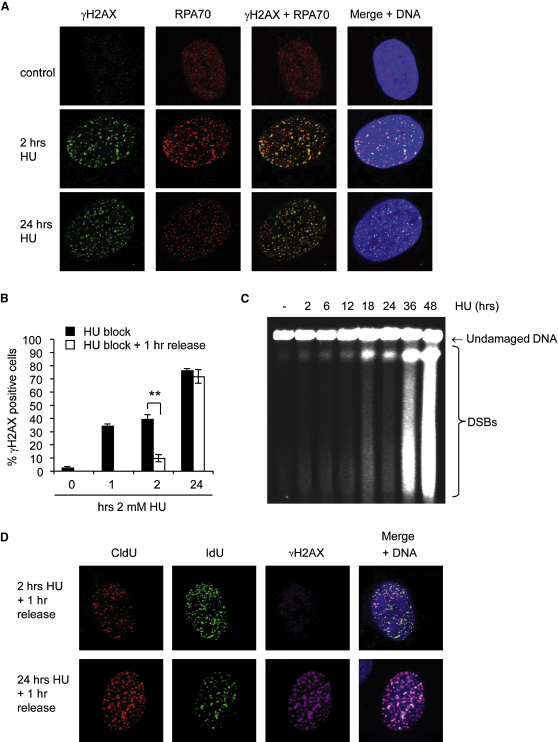
Replication Forks Accumulate DNA Damage during Long Replication Blocks (A) Colocalization of HU-induced γH2AX with RPA. Cells were left untreated or treated with 2 mM HU for 2 or 24 hr, fixed, and immunostained for γH2AX (green) and RPA70 (red). DNA was counterstained with DAPI (blue). (B) Quantification of U2OS cells displaying γH2AX immunostaining after 0, 1, 2, or 24 hr of treatment with 2 mM HU and 1 hr after release from 2 or 24 hr HU treatment. Cells containing more than 10 foci were scored as positive. The means and SD (bars) of three independent experiments are shown. Values marked with asterisks are significantly different (^∗∗^*p* < 0.01). (C) Pulsed-field gel electrophoresis to visualize DSB induction in U2OS cells treated with 2 mM HU for 2 to 48 hr. (D) Colocalization of γH2AX and stalled replication forks in U2OS cells. Cells were pulse-labeled with CldU for 20 min, treated with 2 mM HU for 2 or 24 hr, and released into IdU for 1 hr. Cells were immunostained for CldU (red), IdU (green), and γH2AX (far red), and DNA was counterstained with DAPI (blue). DNA was denatured with HCl to allow CldU/IdU detection.

To confirm the inactivation of replication forks by long HU blocks, we measured the formation of the phosphorylated histone variant H2AX (γH2AX) at inactivated forks. γH2AX accumulates quickly during HU blocks (Figures 2A and 2B), even before DSB induction is observed (Figure 2C) (Saintigny et al., 2001). The γH2AX signal colocalized with RPA foci, suggesting that it marks regions of extensive single-stranded DNA at stalled forks (Figure 2A). We released cells from the HU block for 1 hr and measured how much γH2AX remained after replication had resumed (Figure 2B). We found that γH2AX rapidly disappeared after release from the 2 hr HU block. In contrast, γH2AX foci persisted after release from 24 hr HU block, at times when more DSB were induced (Figure 2B, C). Persisting γH2AX foci colocalized with stalled or inactivated replication forks (Figure 2D). These observations show that DNA damage accumulates at stalled forks with increasing lengths of HU treatments and that this DNA damage persists in cells released from long HU blocks.

### RAD51 Foci and HR Are Induced Late during Replication Blocks

**Figure 3:**
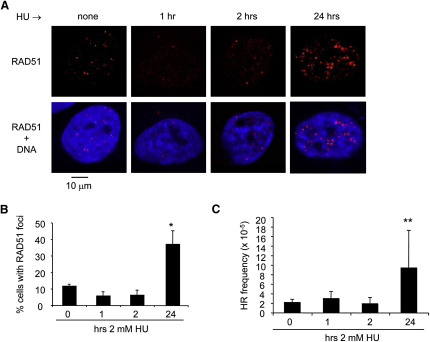
Late Activation of Homologous Recombination during HU Block (A) HU-induced RAD51 foci in U2OS cells. Cells were left untreated or treated with 2 mM HU for 1, 2, or 24 hr, fixed, and immunostained for RAD51 (red). DNA was counterstained with DAPI (blue). (B) Quantification of HU-induced RAD51 foci in U2OS cells. Cells were treated with 2 mM HU for the times indicated and immunostained for RAD51. Cells containing >10 RAD51 foci were scored as positive. The means and range (bars) of two independent experiments are shown. (C) Recombination frequencies in SPD8 cells induced by HU. Cells were treated with 2 mM HU for the times indicated and recovered for 48 hr. HPRT^+^ revertants were quantified 7 days later. The means and SD (bars) of four to ten independent experiments are shown. Values marked with asterisks are significantly different from control (^∗^*p* < 0.05, ^∗∗^*p* < 0.01, and ^∗∗∗^*p* < 0.001; see also Figure S2).

To analyze the response of RAD51 and HR at early and late times of replication blocks, we performed time courses of RAD51 foci formation and HR frequencies induced by HU treatment. Cells were treated with HU for different times, and the percentages of cells containing more than 10 RAD51 foci were quantified. RAD51 foci are induced after 24 hr, but not after 1 or 2 hr HU treatment in U2OS cells (Figures 3A and 3B). To analyze the time course of HR induction by HU treatments, we used the SPD8 cell line, which carries a recombination reporter in the *hprt* gene. HR by either unequal sister chromatid exchange, intrachromatid exchange, single-strand annealing, or gene conversion can lead to restoration of the wild-type *hprt* gene encoding a functional HGPRT protein (Helleday et al., 1998). We found that HR is induced by HU treatments of 24, but not by treatments of 1 or 2 hr (Figure 3C). This finding suggested that HR is not active while replication forks restart, but is induced when replication forks become inactivated. Because we used SPD8 cells to measure HR, we confirmed that they display similar progressive replication fork inactivation as do U2OS cells (Figures S2A–S2D). Forks become inactivated considerably earlier in SPD8 cells, accompanied by earlier RAD51 foci formation (Figure S2E), which colocalize with stalled replication forks (Figure S2F). DSB induction occurred earlier in SPD8 cells as well, although there was no difference to U2OS cells at 2 hr (Figure S2G). These observations confirm that HR activation occurs late during replication blocks and coincides with or is preceded by replication fork inactivation and DSB formation.

### RAD51 Promotes Early Fork Restart

**Figure 4:**
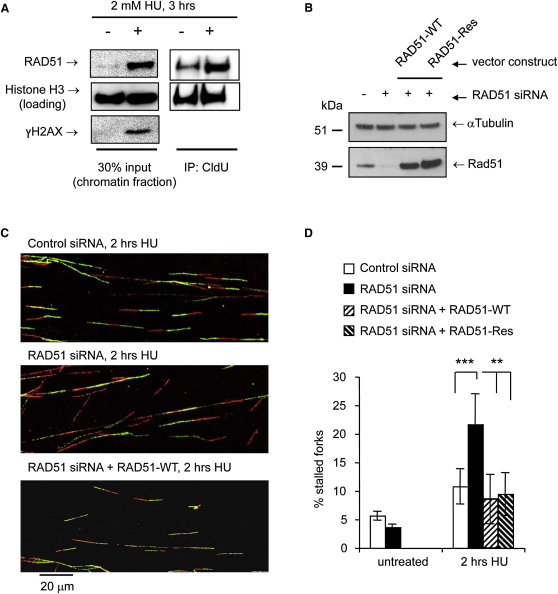
Early Role of RAD51 in Promoting Replication Fork Restart (A) Localization of human RAD51 to replication forks by CldU coimmunoprecipitation (IP). U2OS cells were treated with 2 mM HU for 3 hr, released from HU, and pulse-labeled with CldU for 40 min. Cells were cross-linked, and the chromatin fraction was isolated (input) and subjected to IP using anti-CldU antibody (IP). Fractions were probed for RAD51, γH2AX, and Histone H3 (loading control). (B) Protein levels of RAD51 and α-Tubulin (loading control) in U2OS cells after 48 hr depletion with RAD51 or control siRNA. For re-expression of RAD51, cells were cotransfected with expression constructs encoding wild-type (RAD51-WT) or targeting-resistant (RAD51-Res) RAD51. (C) Representative images of replication tracks after restart from 2 hr HU treatment in control- and RAD51-depleted U2OS cells and RAD51-depleted cells re-expressing RAD51. Untreated values were only determined for control and RAD51 siRNA. (D) Quantification of fork restart in cells as in (C). Stalled replication forks are shown as percentage of all CldU labeled tracks. The means and SD (bars) of at least three independent experiments are shown. Values marked with asterisks are significantly different from control (Student's *t* test, ^∗∗^*p* < 0.01 and ^∗∗∗^*p* < 0.001).

Because RAD51 foci have been suggested to require several kilobases of single-stranded DNA (Raderschall et al., 1999), we tested whether shorter HU treatments lead to a form of RAD51 recruitment not detectable as foci. Coimmunoprecipitation with CldU-labeled replication sites showed that RAD51 localizes to chromatin after short HU treatments in U2OS cells and even in the absence of HU (Figure 4A). These observations suggest that RAD51 and HR might play different early and late roles at stalled replication forks. A possible role of RAD51 during short replication blocks would be to promote the restart of stalled replication forks. To test this idea, we determined the effect of RAD51 depletion on the restart of individual replication forks after HU blocks. We depleted U2OS cells of RAD51 using siRNA (Figure 4B). We found that RAD51 depletion increases the number of forks that do not resume replication after release from 2 hr HU block (Figures 4C and 4D). To confirm specificity of the siRNA treatment, RAD51 siRNA-treated cells were cotransfected with expression vectors encoding wild-type (RAD51-WT) or siRNA targeting resistant (RAD51-Res) RAD51 (Figure 4B). Re-expression of RAD51 protein reduced percentages of stalled forks after release from 2 hr HU treatment back to control levels (Figures 4C and 4D). These data show that RAD51 is required to reactivate a subset of replication forks after short HU blocks.

### XRCC3 Promotes Fork Restart after Short Replication Blocks

**Figure 5:**
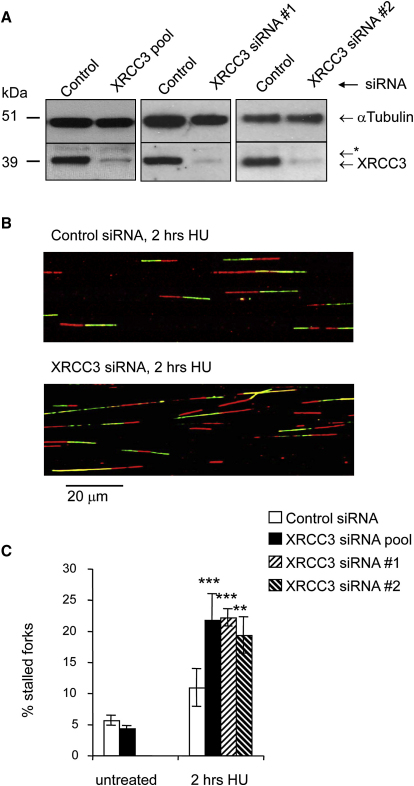
XRCC3 Promotes the Early Restart of Stalled Replication Forks (A) Protein levels of XRCC3 and α-Tubulin (loading control) in U2OS cells after 48 hr depletion with control siRNA, pool of 4 XRCC3 siRNAs, or individual XRCC3 siRNAs #1 and #2. The asterisk denotes a nonspecific band. (B) Representative images of replication tracks after restart from 2 hr HU treatment in control- and XRCC3-depleted U2OS cells. (C) Quantification of fork restart in control- or XRCC3-depleted U2OS cells. The means and SD (bars) of at least three independent experiments are shown. Values marked with asterisks are significantly different from control (Student's *t* test, ^∗∗^*p* < 0.01 and ^∗∗∗^*p* < 0.001).

The XRCC3 protein is required for RAD51 ssDNA complex formation (Bishop et al., 1998). To test whether RAD51 complex formation, which is required for strand invasion (Baumann et al., 1996), is involved in replication restart, we analyzed whether XRCC3 is also required for early restart of replication forks by depleting U2OS cells of XRCC3 using siRNA (Figure 5A). Forty-eight hours later, cells were pulse-labeled with CldU, blocked with HU, and released into IdU for 1 hr. We found that, like RAD51 depletion, XRCC3 depletion increases the number of forks that do not resume replication after release from 2 hr HU block (Figures 5B and 5C). To confirm the specificity of the XRCC3 depletion, we used two individual siRNA duplexes targeting different sequences in the XRCC3 mRNA, with similar results (Figure 5C). These observations demonstrate that, like RAD51, XRCC3 is required to reactivate stalled replication forks.

### The S Phase Checkpoint Suppresses New Origin Firing at Stalled or Collapsed Replication Forks

**Figure 6:**
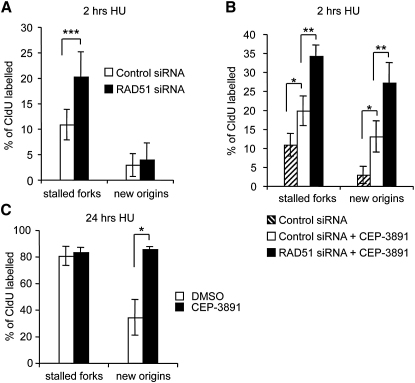
The S Phase Checkpoint Inhibits New Origin Firing Induced by Stalled Replication Forks (A) Quantification of fork restart and new origin firing in control- or RAD51-depleted U2OS cells after release from 2 hr HU treatment. Quantification of fork restart is as in Figure 4D. (B) Quantification of fork restart and new origin firing in U2OS cells after release from 2 hr HU treatment in presence or absence of Chk1 inhibitor CEP-3891 and RAD51 siRNA. CEP-3891 (500 nM) was present throughout HU treatment and during restart. (C) Quantification of fork restart and new origin firing in mock- or CEP-3891-treated U2OS cells after release from 24 hr HU treatment. CEP-3891 (500 nM) was added 1 hr before release from HU block and was present during restart. Replication structures are shown as percentage of all CldU-labeled tracks. The means and SD (bars) of three independent experiments are shown. Values marked with asterisks are significantly different from control (Student's *t* test, ^∗^*p* < 0.05, ^∗∗^*p* < 0.01, and ^∗∗∗^*p* < 0.001; see also Figure S3).

Although reduced fork restart after long HU blocks was accompanied by increased new origin firing, origin firing was not elevated when RAD51-depled cells were released from short HU blocks (Figure 6A). To test whether this might be due to origin suppression by the S phase checkpoint, we analyzed replication restart in the presence or absence of the Chk1 inhibitor CEP-3891 (Figure 6B). We found that Chk1 inhibition alone did reduce fork restart to the same extent as RAD51 depletion, which is in agreement with previous reports that Chk1 stabilizes stalled replication forks (Feijoo et al., 2001; Zachos et al., 2003). Chk1 inhibition also dramatically increased new origin firing after release from 2 hr HU block (Figure 6B). These effects were specific to HU treatment (Figure S3). When RAD51 depletion and Chk1 inhibition were combined, we observed an additive effect on both fork stalling and new origin firing (Figure 6B). This finding suggests that failed fork restart due to RAD51 depletion can trigger new origin firing, but after short HU blocks, the checkpoint prevents this from being a significant mechanism of replication restart. Even after release from 24 hr HU treatment, when new origin firing was increased by 10 fold, Chk1 inhibition during the time of restart increased new origin firing even further (Figure 6C), suggesting that the S phase checkpoint still suppresses a large amount of origin firing after release from long HU blocks. Chk1 inhibitor was only present during the last hour of HU block and did not affect fork restart (Figure 6C).

### RAD51 Promotes Repair of Collapsed Replication Forks after Long Replication Blocks

**Figure 7:**
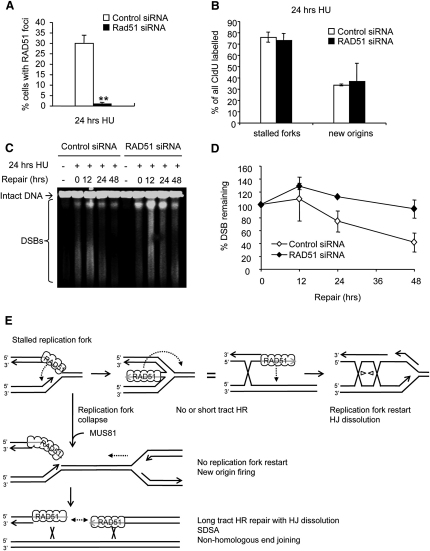
After Long Replication Blocks, RAD51 Foci Do Not Promote Fork Restart but Are Required for DNA Damage Repair (A) Percentage of control- or RAD51-depleted U2OS cells containing more than 10 RAD51 foci after 24 hr treatment with 0.5 mM HU. The means and range (bars) of two independent experiments are shown. Values marked with asterisks are significantly different from control (*p* < 0.01). (B) Fork restart and new origin firing after release from 24 hr treatment with 2 mM HU in control- or RAD51-depleted U2OS cells. The means and SD (bars) of three independent experiments are shown. Replication structures are shown as percentage of all CldU-labeled tracks. (C) Pulsed-field gel electrophoresis to visualize DSB remaining in control- or RAD51-depleted U2OS cells after 0, 12 24, 36, and 48 hr release from 24 hr treatment with 2 mM HU. (D) Quantification of DSB remaining in control- or RAD51-depleted U2OS cells as in (C). The means and range (bars) of two to three independent experiments are shown. (E) Model of RAD51-mediated replication fork restart and repair. RAD51 may have a similar role as recA in *E. coli*, promoting the formation of a Holliday Junction intermediate (chicken foot). The DNA end may then assist to restart replication that would involve recombination over a small area (short tract). Holliday Junction dissolution by the BLM-Top3 complex would dissolve any remaining double Holliday Junctions (Wu and Hickson, 2003). New origin firing rescues replication of collapsed replication forks, which are then repaired by long tract HR involving double Holliday Junction dissolution, synthesis-dependent strand annealing (SDSA) or nonhomologous end joining (see also Figure S4).

Our data suggested that RAD51 promotes replication fork restart after short replication blocks, when forks are still competent to restart. Next, we wanted to analyze the effect of RAD51 on fork restart after long HU blocks, when RAD51 foci accumulate and HR is activated. We confirmed that control cells, but not RAD51-depleted cells, formed RAD51 foci in response to 24 hr HU block (Figure 7A). We used the DNA fiber technique to determine the effect of RAD51 depletion and lack of RAD51 foci on fork restart after 24 hr HU block. We found that, in contrast to release from short HU treatments, RAD51 depletion does not decrease the number of forks that restart after release from long HU blocks (Figure 7B). RAD51 depletion did not affect new origin firing (Figure 7B). However RAD51-depleted cells repair DNA damage induced by long HU treatment less efficiently than do control cells, as demonstrated by the higher amounts of DSB remaining in RAD51-depleted cells up to 48 hr after release from HU block (Figures 7C and 7D). Similarly, a larger number of RAD51-depleted cells still contained γH2AX signal up to 48 hr after release from HU (Figure S4). These observations suggest that there is no correlation between the ability of cells to form RAD51 foci and the ability to restart replication forks. Rather, RAD51 foci formation coincides with replication fork inactivation, DSB formation, and the requirement for RAD51 for DNA repair. Taken together, these data support the idea that RAD51 protein promotes fork restart without forming foci, whereas RAD51 foci formation is a step in the recombination process that repairs collapsed forks.

## Discussion

We have shown that most replication forks resume progression after short replication bocks by HU but do not restart after long HU blocks in either U2OS or SPD8 cells. Our data suggest that stalled replication forks retain the ability to restart for some time before becoming inactivated in a process that coincides with accumulation of DNA damage and DSB formation. In fission yeast, the replication checkpoint controls the structural integrity of stalled replication forks and prevents the formation of recombination foci (Alabert et al., 2009; Barlow and Rothstein, 2009; Meister et al., 2005). The checkpoint also controls MUS81 activity, and HU-stalled forks are processed into DSBs in checkpoint-deficient mutants only (Froget et al., 2008; Kai et al., 2005). In mammalian cells, the ATR- and Chk1-dependent checkpoint keeps replication foci active and prevents excessive DSB formation during replication blocks, suggesting that similar control mechanisms exist (Feijoo et al., 2001; Sorensen et al., 2005; Zachos et al., 2003). Fork collapse in mammalian cells could therefore result from checkpoint adaptation or a leaky checkpoint that allows gradual disintegration or collapse of stalled replication forks. It was recently shown that vertebrate Polo-like kinase 1 mediates adaptation of the replication and G2/M checkpoints (Syljuasen et al., 2006; Yoo et al., 2004) and can also constitutively down-regulate the S phase checkpoint (Trenz et al., 2008). We observe reduced fork restart if Chk1 is inhibited during short HU blocks, supporting the idea that the S phase checkpoint stabilizes stalled replication forks. However, checkpoint inhibition during a 2 hr HU treatment inactivates a much smaller number of forks, compared with a 24 hr HU treatment, suggesting that the length of the replication block plays an important role in fork inactivation. Cleavage of forks into DSBs does not seem to be the primary cause of fork inactivation, because MUS81 depletion did not affect fork restart in U2OS cells, and fork inactivation after short HU blocks is much higher in SPD8 cells, without more DSB formation. However, fork cleavage into DSBs might be a result of fork inactivation and prerequisite for replication fork repair.

It has been proposed that eukaryotic forks might not necessarily need to restart because replication can be completed from adjacent origins (Branzei and Foiani, 2007; Paulsen and Cimprich, 2007). We now show that new origin firing can indeed be a major mechanism by which mammalian cells resume replication. Stalled forks do not need to be converted into DSBs to trigger new origin firing, because MUS81-depleted cells also display increased origin firing after release from long HU blocks. This finding is in agreement with previous reports that globally reduced replication fork speeds alone can increase origin firing (Anglana et al., 2003; Ge et al., 2007). Ge and co-workers (2007) also showed that replication inhibition can trigger new origin firing locally within active replication clusters. However, our data suggest that, after short HU treatments, any large-scale increase in new origin firing is prevented by the Chk1-mediated S phase checkpoint. This also applies to new origin firing after direct fork collapse induced by camptothecin (Seiler et al., 2007 and data not shown). However, after long HU treatments, suppression of new origin firing by the checkpoint is clearly incomplete, which may lend some support to the idea that checkpoint adaptation occurs. Alternatively or in addition, the sheer extent of replication fork inactivation could allow so many dormant origins to fire that a larger number can escape the checkpoint.

Our observations that replication forks do not restart after prolonged HU treatments are in disagreement with previously published observations observing fork restart after 24 hr HU (Hanada et al., 2007). These data were obtained using mouse embryonic stem cells, and we cannot exclude differences between different cell systems; it is noteworthy that both U2OS and SPD8 are transformed cell lines. Interestingly, it has been reported that p53 protects cells from HU-induced DSBs (Kumari et al., 2004), which agrees with the different kinetics of fork inactivation in p53-proficient U2OS and p53-deficient SPD8 cells.

In *E. coli*, Holliday Junctions are formed as intermediates for restart of stalled replication forks (McGlynn and Lloyd, 2002). RecA, the RAD51 homolog in *E. coli*, is required for the formation of these Holliday Junctions in the absence of dnaB (Seigneur et al., 2000). Here, we suggest that RAD51 plays a similar role in mammalian cells. In such a model, RAD51 would coat ssDNA regions occurring surrounding a stalled replication fork and would then invade the homologous molecule, which would facilitate the formation of a Holliday Junction, often referred to as a “chicken foot” (Figure 7E). RAD51 could stabilize this structure by binding the ssDNA tail of paired nascent strands or possibly the dsDNA of the reversed fork, if this is not prevented by BRCA2 (Carreira et al., 2009). Such a chicken foot structure may be more stable and may facilitate replication restart from the DNA end, which could potentially also be mediated by RAD51-dependent strand invasion. This type of end-induced replication should theoretically be able to cause a recombination event in a recombination reporter. To detect recombination in this reporter requires an unequal recombination event occurring over several kilobases of DNA, and the recombination tract involved in restarting a replication fork is likely restricted to a very short piece of DNA because the Holliday Junction would prevent recombination at a distant site. The benefit of RAD51-mediated restart of forks stalled by replication inhibitors might be that the Holliday Junction intermediate can serve as a substrate for origin-independent replisome loading, as appears to be the case in bacteria (Heller and Marians, 2006).

Our data suggest that the HR induced by HU (Lundin et al., 2002; Saintigny et al., 2001) does not restart replication forks but performs postreplication repair of collapsed forks. In line with this argument, a recent report showed that RAD51-dependent mechanisms remove spontaneously accumulated ssDNA foci preferentially during the G2 phase of the cell cycle (Su et al., 2008), suggesting that HR is temporally separated from DNA replication that creates these foci. It has been previously shown that HR also preferentially repairs direct DSBs during late S/G2 phase (Rothkamm et al., 2003; Takata et al., 1998; Vispe et al., 1998), but this observation could be due to the fact that HR can repair DSBs only if two sister chromatids are present, which is more likely after replication has been completed. If a replication fork from an adjacent origin arrived at an unrepaired collapsed fork, this would result in a two-ended DSB that could also be a substrate for nonhomologous end joining. This would explain the HU sensitivity of nonhomologous end joining mutant cells previously reported (Lundin et al., 2002; Saintigny et al., 2001).

Taken together, our data suggest that RAD51 has distinct early and late roles during replication blocks and promotes the restart of stalled forks and the repair of collapsed forks by different mechanisms.

## Experimental Procedures

### Cell Lines and Reagents

U2OS cells were obtained from ATCC. SPD8 cells have been described elsewhere (Helleday et al., 1998). Cells were confirmed to be free of *Mycoplasma* infection and grown in Dulbecco's modified Eagle's Medium with 10% fetal bovine serum in a humidified atmosphere containing 5% CO_2_. CEP-3891 was obtained from Cephalon.

### DNA Fiber Analysis

U2OS cells were pulse-labeled with 25 μM CldU for 20 min, washed three times with medium, incubated in 2 mM HU for times indicated, washed three times with medium, and pulse-labeled with 250 μM IdU for 1 hr. Labeled cells were harvested, and DNA fiber spreads were prepared as described elsewhere (Henry-Mowatt et al., 2003). CldU was detected by incubating acid-treated fiber spreads with rat anti-BrdU monoclonal antibody (1:1000; AbD Serotec) for 1 hr. Slides were fixed with 4% PFA and incubated with AlexaFluor 555–conjugated goat anti–rat IgG (1:500; Molecular Probes) for 1.5 hr. IdU was detected using mouse anti-BrdU monoclonal antibody (1:1000; Becton Dickinson) overnight at 4°C and AlexaFluor 488–conjugated goat anti–mouse IgG (1:500; Molecular Probes) for 1.5 hr. Fibers were examined using a Biorad Radiance confocal microscope with a 60× oil immersion objective. For quantification of replication structures, at least 250 structures were counted per experiment. The lengths of red (AF 555) or green (AF 488) labeled patches were measured using the ImageJ software (National Institutes of Health; http://rsbweb.nih.gov/ij/) and arbitrary length values were converted into micrometers using the scale bars created by the microscope.

### Immunofluorescence

Primary antibodies were rabbit polyclonal anti-RAD51 (H92, Santa Cruz Biotechnology, 1:500–1:1000), rabbit polyclonal anti-RPA70 (a kind gift from Prof. Rolf Knippers, Konstanz, Germany; 1:1000), mouse monoclonal and rabbit polyclonal anti–phospho-Histone H2AX (Ser139) (both Upstate Biotechnology; 1:1000), rat monoclonal anti-BrdU (AbD Serotec; 1:400) to detect CldU, and mouse monoclonal anti-BrdU (1:50; Becton Dickinson) to detect IdU. Secondary antibodies were anti–rabbit IgG AlexaFluor 555 or AlexaFluor 647, anti–mouse IgG AlexaFluor 488, and anti–rat IgG AlexaFluor 555 (Molecular Probes). For colocalization with replication forks, primary and secondary antibodies against phospho-Histone H2AX were fixed for 10 min with 2% PFA before DNA denaturation with 2 M HCl for 40 min and immunostaining for thymidine analogs. DNA was counterstained with DAPI.

### Pulsed-Field Gel Electrophoresis

Cells (2.5 × 10^6^) were treated with 2 mM HU for the times indicated. For DSB repair experiments, U2OS cells were transfected with control or RAD51 siRNA for 36 hr, then treated with 2 mM HU for 24 hr, and released into fresh medium to allow repair. Afterward, cells were trypsinized and melted into 1.0% InCert-Agarose (BMA) inserts. Subsequently, agarose inserts were digested in 0.5 M EDTA-1% *N*-laurylsarcosyl-proteinase K (1 mg/ml) at 50°C for 48 hr and washed four times in TE buffer. The inserts were loaded onto a separation gel (1.0% chromosomal-grade agarose; Bio-Rad). Separation was performed on a CHEF DR III equipment (BioRad; 120 field angle, 240 s switch time, 4 V cm^−1^, 14°C) for 24 hr. Gels were stained with ethidium bromide, and DSBs were quantified (chromosome fragments >2 Mbp). Densitometric analysis was performed using the PCBASS 2.0 software.

### Recombination in SPD8 Cells

SPD8 cells were grown in the presence of 6-thioguanine to suppress spontaneous recombination. Cells (1.5 × 10^6^) cells were treated with 0.5 mM HU for times indicated and recovered in medium for 48 hr. HPRT^+^ revertants were selected by plating 3 × 10^5^ cells in the presence of HAsT (50 μM hypoxanthine, 10 μM L-azaserine, and 5 μM thymidine). To determine cloning efficiency, two dishes were plated with 500 cells each. Colonies were stained with methylene blue following 7 (for cloning efficiency) or 10 (for reversion) days of incubation (see Supplemental Data for more detailed protocol).

### CldU Coimmunoprecipitation of Proteins Present at Stalled Replication Forks

U2OS cells (2 × 10^6^) were treated with 2 mM HU for 3 hr. HU was removed, and cells were labeled with 100 μM CldU for 40 min. Cells were cross-linked in 1% PFA for 15 min. The cytoplasmic protein fraction was removed by incubation in hypotonic buffer (10 mM HEPES [pH 7], 50 mM NaCl, 0.3 M sucrose, 0.5% TX-100, and protease inhibitor cocktail [Roche]) for 10 min on ice and centrifugation at 1500 g for 5 min. Nuclear soluble fraction was removed by incubation with nuclear buffer (10 mM HEPES [pH 7], 200 mM NaCl, 1 mM EDTA, 0.5% NP-40, and protease inhibitor cocktail) for 10 min on ice and centrifugation at 13,000 rpm for 2 min. Pellets were resuspended in lysis buffer (10 mM HEPES [pH 7], 500 mM NaCl, 1 mM EDTA, 1% NP-40, and protease inhibitor cocktail), sonicated, and centrifuged for 30 s at 13,000 rpm, and the supernatant was transferred to a new tube. Total protein (150 μg) was used for IP with 2 μg anti-CldU antibody (rat-anti-BrdU; OBT0030F AbD Serotec) and 20 μl of Protein A/G-PLUS agarose (Santa Cruz Biotechnology). The IP reaction was washed twice with nuclear buffer and twice with washing buffer (10 mM HEPES and 0.1mM EDTA protease inhibitor cocktail), incubated in 2× sample loading buffer (100 mM Tris HCl [pH 6.8], 100 mM DTT, 4% SDS, 0.2% bromophenol blue, and 20% glycerol) for 30 min at 90°C, and was used for Western Blot with rabbit polyclonal anti-Rad51 (H92, Santa Cruz Biotechnology; 1:500), rabbit polyclonal anti-H3 (Fl-136, Santa Cruz Biotechnology; 1:500), and mouse monoclonal anti-γH2AX (3F2, Abcam; 1:2000).

### siRNA Treatment

siRNA against human Rad51 (Ito et al., 2005), XRCC3 (siGENOME SMARTpool D-012067), and MUS81 (siGENOME SMARTpool D-016143) were purchased from Dharmacon. Individual XRCC3 siRNA #1 and #2 were Dharmacon siGenome D-012067-01 and −04. “Allstars negative control siRNA” was purchased from QIAGEN. Cells were transfected with 50 nM siRNA using Dharmafect 1 reagent (Dharmacon) or Lipofectamine2000 (Invitrogen) for pulsed-field gel electrophoresis. Cells were cultured for 48 hr prior to DNA labeling and HU treatments. Depletion was confirmed by Western Blot using rabbit anti-Rad51 (1:1000; H-92, Santa Cruz), rabbit anti-XRCC3 (1:5000; Novus Biologicals), mouse anti-MUS81 (1:500; MTA30 2G10/3, Santa Cruz), and mouse anti-αTubulin (1:5000; Sigma). For re-expression of Rad51, 50 nM RAD51 siRNA and 78 pM (1 μg) of RAD51 pcDNA3.1/V5/His-TOPO construct (Sorensen et al., 2005) were cotransfected using Lipofectamine2000 (Invitrogen) 48 hr before labeling (see Supplemental Data for more detailed protocol).

### Statistical Analysis

The means and standard deviations of two to ten independent repeats are shown. Error bars are 1× standard deviation. Statistical significance of differences between means was determined using the Student's t test (one-tailed and paired, or using two-sample with equal variance for unpaired arrays).

## Supplemental Information

Document S1. Supplemental Experimental Procedures and Five Figures
