# Supplementary material for: Hydroxyurea-Stalled Replication Forks Become Progressively Inactivated and Require Two Different RAD51-Mediated Pathways for Restart and Repair
Source: Mol Cell. 2010 Feb 26;37(4):492–502. doi: 10.1016/j.molcel.2010.01.021 (PMC2958316; doi:10.1016/j.molcel.2010.01.021)
Supplement: Document S1. Supplemental Experimental Procedures and Five Figures [file mmc1.pdf]

Molecular Cell, ***Volume 37***

Supplemental Information

Hydroxyurea-Stalled Replication Forks Become Progressively Inactivated and Require Two Different RAD51-Mediated Pathways for Restart and Repair

Eva Petermann, Manuel Luís Orta, Natalia Issaeva, Niklas Schultz, and Thomas Helleday

Supplementary Experimental Procedures

Immunofluorescence

$4.5x10^4$ VC8-B2 cells were grown on coverslips overnight and treated with HU as indicated. Cells were fixed with paraformaldehyde and permabilised with 0.3% Triton-X 100. Coverslips were incubated with rabbit polyclonal anti-Rad51 (H92, Santa Cruz Biotechnology, 1:500-1:1000). For BrdU and Rad51 colocalisation, cells were treated with 20 μM BrdU for 5 minutes before 0.5 mM HU was added for 4 hours. Cells were fixed and treated with rabbit polyclonal anti-Rad51 (H92, Santa Cruz Biotechnology, 1:500), then fixed for 10 minutes with 2% PFA. Coverslips were treated with 2M HCl for 40 minutes, rinsed with sodium borate buffer pH 8.7, and incubated with mouse monoclonal anti-BrdU antibody (Becton Dickinson, 1:50) to detect BrdU. DNA was counterstained with ToPro (Molecular Probes). Secondary antibodies were anti-rabbit IgG conjugated with AlexaFluor 555 and anti-mouse IgG conjugated with AlexaFluor 488 (Molecular Probes).

siRNA treatment

siRNA against MUS81 (siGENOME SMARTpool D-016143) was purchased from Dharmacon. “Allstars negative control siRNA” was purchased from Qiagen. Cells were transfected with 50nM siRNA using Dharmafect 1 reagent (Dharmacon) and cultured for 48h prior to DNA labelling. Depletion was confirmed by Western Blot using mouse anti-MUS81 (1:500, MTA30 2G10/3, Santa Cruz) and mouse anti-αTubulin (1:5000, Sigma).

siRNA targeting-resistant RAD51 expression constructs

The siRNA targeting-resistant RAD51 expression construct RAD51-Res pcDNA3.1/V5/His-TOPO was generated by site-directed mutagenesis (Quikchange, Stratagene) of RAD51-WT pcDNA3.1/V5/His-TOPO using appropriate oligonucleotides to introduce three silent mutations (underlined): 5'- TGGCTCCAAAGAGCTTGACAA<u>G</u>CTACT<u>G</u>CA<u>G</u>GGTGGAATTGAGACTG-3' and 5'-CAGTCTCAATTCCACC<u>C</u>TG<u>C</u>AGTAG<u>C</u>TTGTCAAGCTCTTTGGAGCCA-3'

Recombination in SPD8 cells

SPD8 cells were grown in the presence of 5 µg/ml 6-thioguanine to suppress spontaneous recombination. 1.5×10^6 cells were inoculated into 100mm dishes in medium 4 hours prior to a 24-hour treatment with drugs as indicated. Short treatments were made next day. After treatments, the cells were rinsed three times with PBS and 20ml medium added before allowing the cells to recover for 48 h. After recovery, cells were released by trypsinisation and counted. $HPRT^+$ revertants were selected by plating 3×10^5 treated cells per dish in the presence

of HAsT (50 μM hypoxanthine, 10 μM L-azaserine, and 5 μM thymidine). To determine cloning efficiency, two dishes were plated with 500 cells each. The colonies obtained were stained with methylene blue in methanol (4 g/L), following 7 (in the case of cloning efficiency) or 10 (for reversion) days of incubation.

Supplemental Figures

Fig S1, related to Figure 1. Replication fork progression during 24 hours HU block in U2OS cells, and fork restart and new origin firing after release from 24 hours HU treatment in MUS81 depleted cells. (A) Labelling protocol for DNA fibre analysis of replication forks. U2OS cells were pulse labelled with CldU, and then treated with HU for 24 hours in presence of IdU.

(B) Representative image of replication tracks after 24 hours HU treatment in U2OS cells.

(C) Quantification of distances moved by replication forks during 24 hours HU block. The third quartile track length indicates that 75% of all forks were shorter than 5.7 +/- 2.7 μm.

(D) Protein levels of MUS81 and α-Tubulin (loading control) in U2OS cells after 72 hours depletion with MUS81 or control siRNA.

(E) Representative images of replication tracks after restart from 24 hours HU treatment in control- and MUS81-depleted U2OS cells.

(F) Quantification of fork restart and new origin firing in cells as in (B). Replication structures are shown as percentage of all CldU labelled tracks. The means and S.D. (bars) of three independent experiments are shown

Fig S2, related to Figure 3. Replication restart and replication fork progression during HU treatment in SPD8 cells. (A) Representative images of replication tracks after release from 2 or 24 hours HU treatment in SPD8 hamster cells.

(B) Quantification of fork restart after release from 2 or 24 hours HU treatment in SPD8 cells.

(C) SPD8 cells were pulse labelled with CldU, and then treated with HU for 24 hours in presence of IdU. Representative image of replication tracks after 24 hours HU treatment are shown.

(D) Quantification of distances moved by replication forks during 24 hours HU block. The third quartile track length indicates that 75% of all forks were shorter than 2.1 +/- 0.3 μm.

(E) HU-induced RAD51 foci in VC8-B2 hamster cells. Cells were treated with 0.5 mM HU for the times indicated and immunostained for RAD51. Cells containing > 10 RAD51 foci were counted as positive.

(F) Co-localisation of RAD51 and replication foci in HU-treated VC8-B2 cells. Cells were labelled with 20 μM BrdU for 5 minutes, then treated with 0.5 mM HU for 4 hours, and immunostained for RAD51, BrdU and DNA. DNA was denatured with HCl to allow BrdU detection.

(G) Pulse-field gel electrophoresis to visualise DSB induction in SPD8 cells treated with 2 mM HU for the times indicated.

The means and S.D. (bars) of three independent experiments are shown. Values marked with asterisks are significantly different from control (* $p < 0.05$, ** $p < 0.01$).

Fig S3, related to Figure 6. Effect of Chk1 inhibition on replication fork restart and new origin firing in presence and absence of HU. (A) Quantification of fork stalling in U2OS cells treated with Chk1 inhibitor CEP-3891 (500 nM) or DMSO alone or treated with CEP-3891 or DMSO during 2 hours HU block.

(B) Quantification of new origin firing in U2OS cells treated as in (A).

The means and S.D. (bars) of three independent experiments are shown. Values marked with asterisks are significantly different from control (student's t-test, * $p < 0.05$, ** $p < 0.01$).

Fig. S4, related to Figure 7. γH2AX staining in control- or RAD51-depleted U2OS cells released from 24 hours HU treatment. (A) Percentage of γH2AX remaining in control- or RAD51-depleted U2OS cells after 0, 12 24, 36 and 48 hours release from 24 hours treatment with 2 mM HU. Cells containing more than 10 foci were scored as positive. The means and S.D. (bars) of three independent experiments are shown.

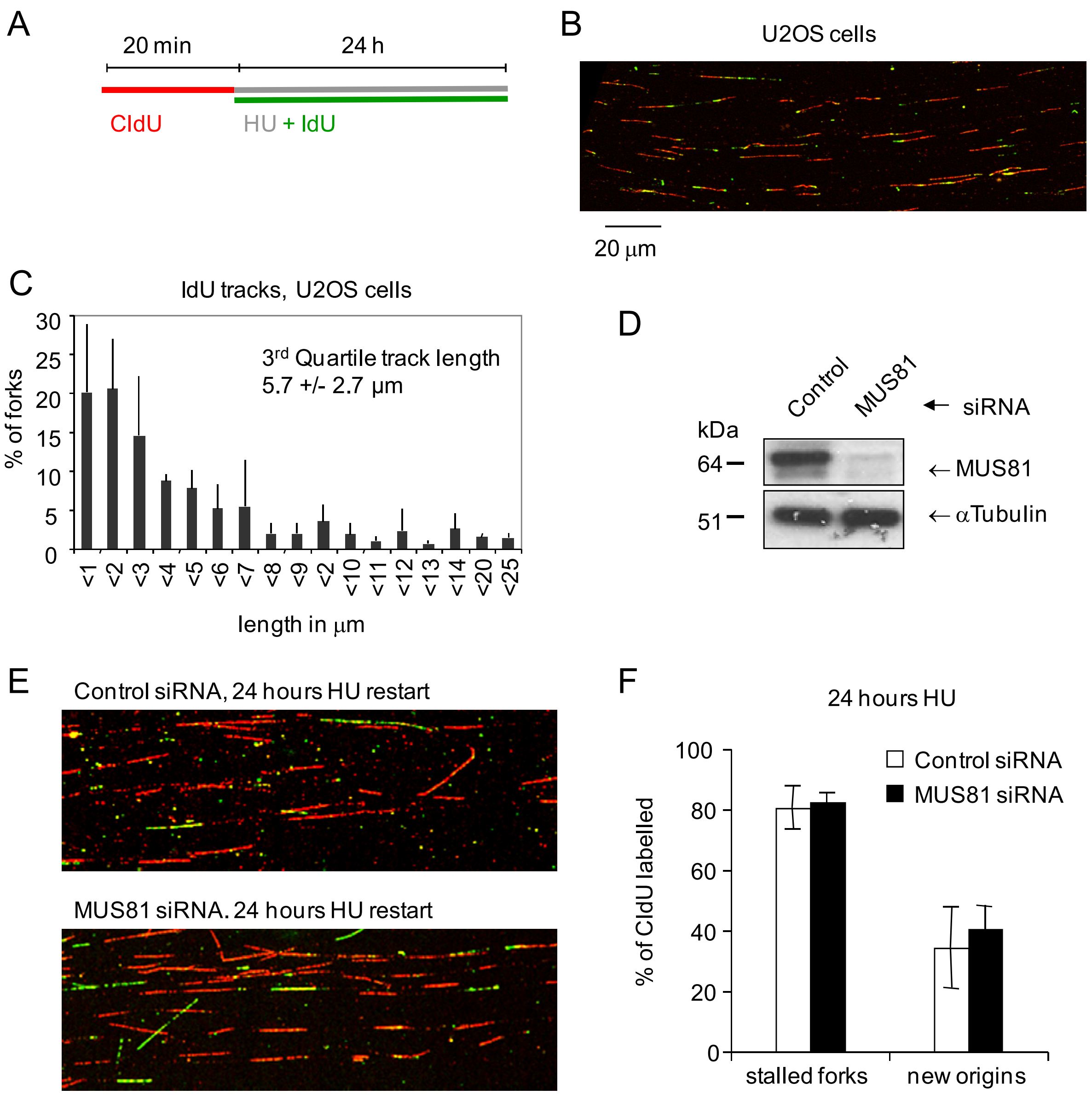

A
20 min
24 h
CldU
HU + IdU
B
U2OS cells
20 µm
C
IdU tracks, U2OS cells
3rd Quartile track length
5.7 +/- 2.7 µm
% of forks
30
25
20
15
10
5
0
<1
<2
<3
<4
<5
<6
<7
<8
<9
<2
<10
<11
<12
<13
<14
<20
<25
length in µm
D
Control
MUS81
siRNA
kDa
64
51
MUS81
αTubulin
E
Control siRNA, 24 hours HU restart
MUS81 siRNA. 24 hours HU restart
F
24 hours HU
% of CldU labelled
100
80
60
40
20
0
Control siRNA
MUS81 siRNA
stalled forks
new origins

Figure S1

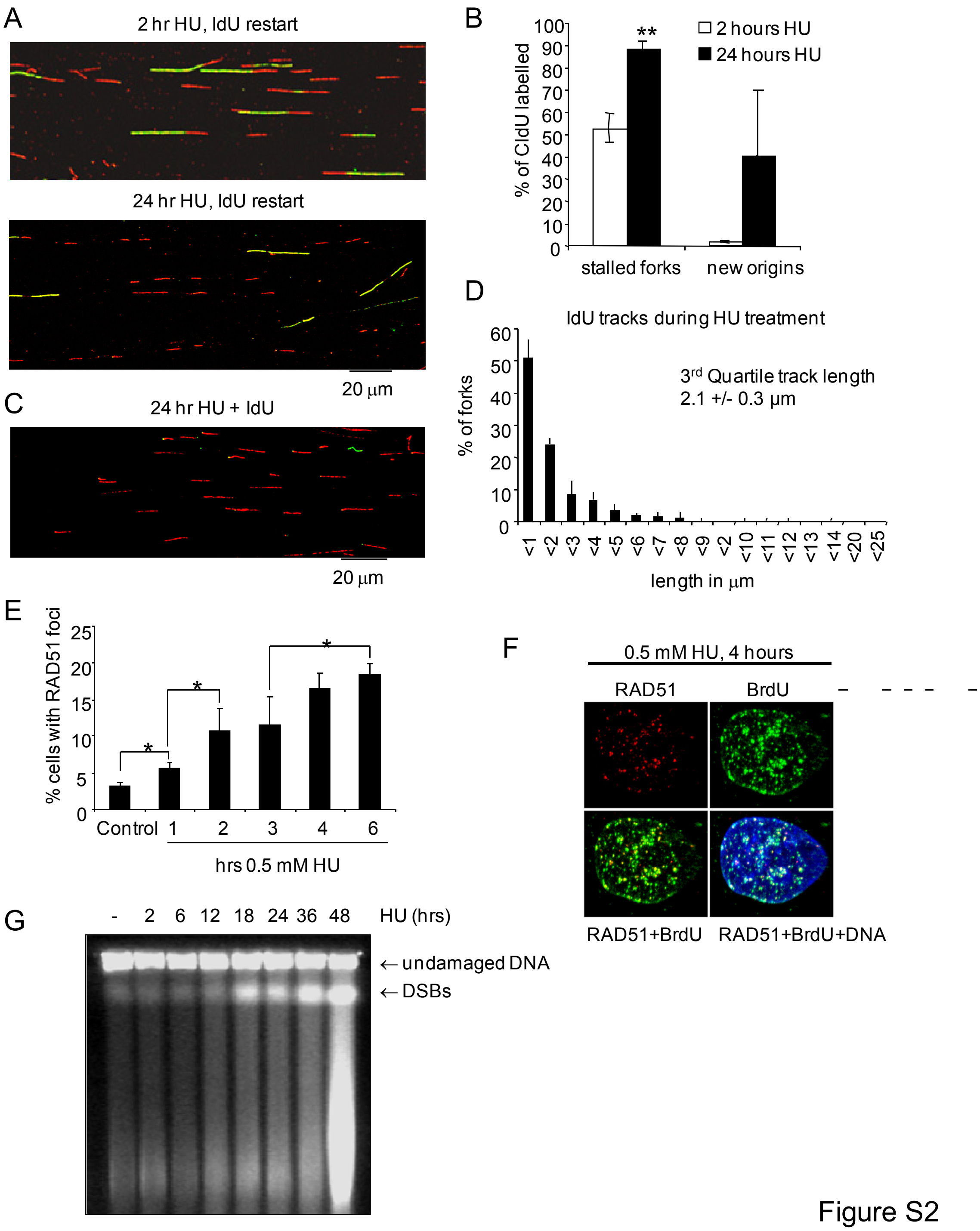
A
2 hr HU, IdU restart
24 hr HU, IdU restart
20 μm
B
% of CldU labelled
100
90
80
70
60
50
40
30
20
10
0
**
2 hours HU
24 hours HU
stalled forks
new origins
C
24 hr HU + IdU
20 μm
D
IdU tracks during HU treatment
% of forks
60
50
40
30
20
10
0
3rd Quartile track length
2.1 +/- 0.3 μm
<1 <2 <3 <4 <5 <6 <7 <8 <9 <2 <10 <11 <12 <13 <14 <20 <25
length in μm
E
% cells with RAD51 foci
25
20
15
10
5
0
*
*
*
Control 1 2 3 4 6
hrs 0.5 mM HU
F
0.5 mM HU, 4 hours
RAD51
BrdU
RAD51+BrdU
RAD51+BrdU+DNA
G
- 2 6 12 18 24 36 48
HU (hrs)
← undamaged DNA
← DSBs

Figure S2

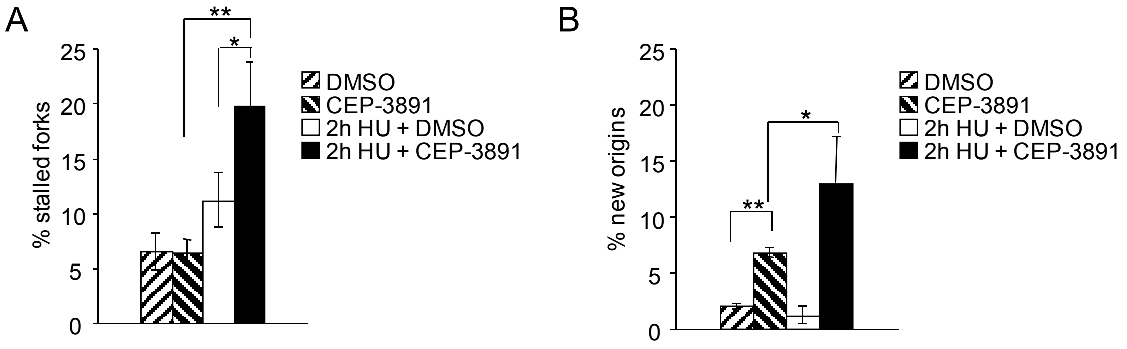
A
**
*
25
20
15
10
5
0
% stalled forks
DMSO
CEP-3891
2h HU + DMSO
2h HU + CEP-3891
B
25
20
15
10
5
0
% new origins
*
**
DMSO
CEP-3891
2h HU + DMSO
2h HU + CEP-3891

Figure S3

A

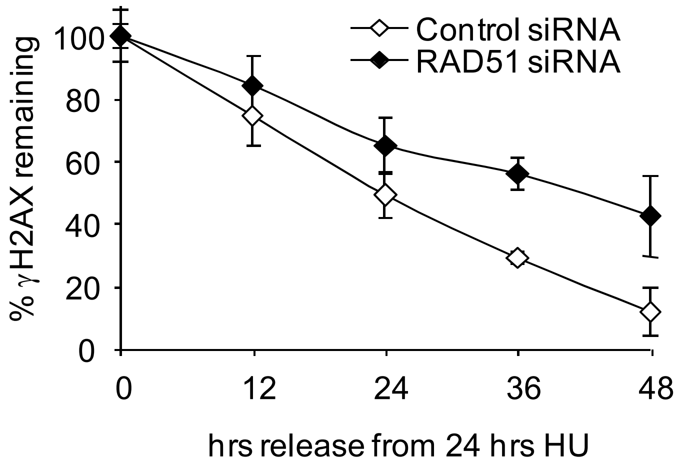

Control siRNA
RAD51 siRNA
% γH2AX remaining
100
80
60
40
20
0
0
12
24
36
48
hrs release from 24 hrs HU

Figure S4
